# Malaria Control, Elimination, and Prevention as Components of Health Security: A Review

**DOI:** 10.4269/ajtmh.22-0038

**Published:** 2022-09-06

**Authors:** Ruwanthi Perera, Rajitha Wickremasinghe, Gretchen Newby, Amandhi Caldera, Deepika Fernando, Kamini Mendis

**Affiliations:** ^1^ Department of Public Health, Faculty of Medicine, University of Kelaniya, Ragama, Sri Lanka;; ^2^ Malaria Elimination Initiative, Institute for Global Health Sciences, University of California–San Francisco, San Francisco, California;; ^3^ Department of Parasitology, Faculty of Medicine, University of Colombo, Colombo, Sri Lanka

## Abstract

International travel, a major risk factor for imported malaria, has emerged as an important challenge in sustaining malaria elimination and prevention of its reestablishment. To make travel and trade safe, the WHO adopted the International Health Regulations (IHR) which provides a legal framework for the prevention, detection, and containment of public health risks at source. We conducted a systematic review to assess the relevance and the extent of implementation of IHR practices that can play a role in reducing malaria transmission. Selected studies addressed control, elimination, and prevention of reestablishment of malaria. Study themes focused on appraisal of surveillance and response, updating national policies to facilitate malaria control and elimination, travel as a risk factor for malaria and risk mitigation methods, vector control, transfusion malaria, competing interests, malaria in border areas, and other challenges posed by emerging communicable diseases on malaria control and elimination efforts. Review results indicate that malaria has not been prioritized as part of the IHR nor has the IHR focused on vector-borne diseases such as malaria. The IHR framework in its current format can be applied to malaria and other vector-borne diseases to strengthen surveillance and response, overcome challenges at borders, and improve data sharing—especially among countries moving toward elimination—but additional guidelines are required. Application of the IHR in countries in the malaria control phase may not be effective until the disease burden is brought down to elimination levels. Considering existing global elimination goals, the application of IHR for malaria should be urgently reviewed and included as part of the IHR.

## INTRODUCTION

Infectious diseases travel with people across borders and may spread globally within a short period of time. The International Health Regulations (IHR), adopted by the WHO in 1969 as part of international law, provides a legal framework for the prevention, detection, and containment of public health risks at the source, before they spread across borders, through the collaborative actions of States Parties and the WHO and are intended to make travel and trade safer.^1^^,^^2^

The IHR were extensively revised in 2005, encompassing an all-hazard model applicable to all public health risks (natural, technological, and societal), and came into effect in June 2007.^2^ The scope of the IHR is broad and inclusive to maximize the probability that all such events that could have serious international consequences are identified early and promptly reported by States Parties to WHO for assessment.^3^ The responsibility for implementing the IHR rests jointly on states and the WHO. Independent states are required to develop their national surveillance and response systems to deliver a number of specific core capacities, as well as other notification and reporting requirements at community, intermediate, and national levels.

Malaria is a major public health problem globally, affecting an estimated 241 million people and causing 627,000 deaths in 2020.^4^ This high burden of disease exists despite more than a century of global efforts and research aimed at improving the prevention, diagnosis, and treatment of malaria.^5^ Parasite resistance to drugs and increased mosquito resistance to insecticides have become the most critical challenges in controlling and eliminating malaria in recent years. Mass migration of humans and disease vectors complicate local transmission patterns and present additional challenges for malaria programs.^6^ To make progress toward malaria eradication, the WHO set a goal in 2015 in its Global Technical Strategy for Malaria 2016–2030 (GTS) to reduce the global malaria burden by 90% by 2030. Achieving this goal requires that states make an effort to reduce transmission and reach national elimination goals within their respective countries; those states that have already eliminated malaria must maintain malaria-free status by preventing its reestablishment.^7^

Successful implementation of the IHR will help reach the malaria burden reduction and elimination goals highlighted in the GTS.^7^ However, most of the countries with ongoing malaria transmission are low-income countries, and although the IHR were introduced primarily to ensure capacity building at the national level to achieve the collective goal of global health security,^8^^,^^9^ many low-income countries are unable to implement the necessary core capacities.^10^

We conducted a systematic review to assess the relevance and to describe the implementation of IHR practices that can play a role in reducing malaria transmission and achieving malaria elimination. We specifically focus on implementation of measures to prevent malaria transmission across borders, both local and international.

## METHODS

The protocol was registered on February 11, 2021, in the International Prospective Register of Systematic Reviews (PROSPERO), registration number CRD42021230786. The main outcome was types of health security measures related to malaria that are implemented by countries. Key words used were “health security,” “malaria,” “international health regulations,” “IHR,” “malaria prevention,” and “prevention.” To obtain the maximum number of relevant articles, the searches were carried out with different keyword combinations (Table 1). All study types were included in the review. Searches were not restricted by the date of publication. However, in the EBSCO search, a “journal articles only” filter was used and in OpenGrey the “English results only” filter was used to limit the number of hits.

**Table 1:** Searches done using different databases

Source	Search string	Number of hits
PubMed	“Health security” AND “malaria”	179
	“International health Regulations” OR “IHR” AND “malaria”	56
	“Prevention of Re-Establishment of Malaria”	30
EBSCO	“Health security” AND “malaria”	235
	“International Health regulations” AND “Malaria”	98
OpenGrey	“Malaria prevention” LANG: EN	17
Google Scholar	“health security” OR “international disease regulations” AND “malaria prevention”	393

The titles were screened, and 620 articles that were duplicates or clearly irrelevant were eliminated. In addition, 388 abstracts were screened and 320 that were books, nonscholarly articles, systematic reviews, and non-English articles were eliminated. Selected articles were subjected to a full text review. Data related to the type of health security measures carried out, their effectiveness, problems related to the measures and recommendations were extracted. Two investigators carried out the full text review. A third reviewer was consulted when consensus could not be achieved.

## RESULTS

**Figure 1.**
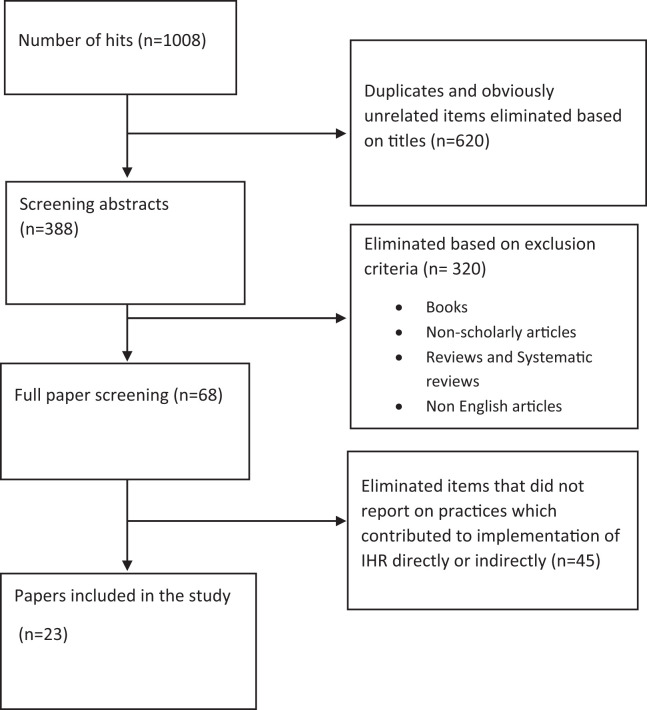
PRISMA flow chart.

The database search was carried out from November 2020 to January 2021, and a total of 23 manuscripts were included in the review (Figure 1).

The included studies encompass a wide variety of settings including countries in Asia Pacific, the Eastern Mediterranean, Africa, and Europe, and addressed control, elimination, and prevention of reestablishment of malaria. The themes of the articles were focused around appraisal of surveillance and response,^11^^–^^18^ updating national policies to facilitate malaria control efforts,^17^^,^^19^^–^^21^ travel as a risk factor for malaria and methods to mitigate the risk,^12^^,^^13^^,^^20^^,^^22^^–^^26^ vector control,^12^^,^^16^^,^^27^ transfusion malaria,^21^^,^^28^ competing interests,^27^^,^^29^^,^^30^ malaria in border areas,^15^^,^^19^^,^^22^^,^^31^ and other challenges posed by emerging communicable diseases on malaria elimination efforts^32^^,^^33^ (Table 2).

**Table 2:** List of studies included in the review

Study	Country(ies)/ Region	Phase of malaria control	Topics Addressed
ACTwatch Group et al.^19^	Myanmar, Lao PDR	Elimination	National malaria policies Border areas
Adhikari et al.^29^	Nepal	Elimination	Competing interests
Ajayi et al.^32^	Related to Africa	Control	Other emerging communicable diseases
Alotaibi et al.^11^	Saudi Arabia	Prevention of reintroduction	Surveillance response
Al Zahrani et al.^22^	Saudi Arabia–Yemen border	Elimination	Travel as a risk factor Border areas
Chen^23^	Taiwan	Prevention of reintroduction	Travel as a risk factor
Danis et al.^12^	Greece	Prevention of reintroduction	Surveillance response Travel as a risk factor Vector control
Fernando et al.^30^	Sri Lanka	Elimination	Competing interests
Fernando et al.^24^	Sri Lanka	Prevention of reintroduction	Travel as a risk factor
Galappaththy et al.^13^	Sri Lanka	Prevention of reintroduction	Surveillance response Travel as a risk factor
Inkochasan et al.^20^	Greater Mekong Subregion countries	Control	National malaria policies Travel as a risk factor
Karunasena et al.^14^	Sri Lanka	Prevention of reintroduction	Surveillance response
Kihika et al.^25^	Kenya	Control	Travel as a risk factor
Kounnavong et al.^21^	Lao PDR	Control	National malaria policies Transfusion malaria
Liverani et al.^15^	Cambodia and Vietnam	Elimination	Surveillance response Border areas
Marasinghe et al.^16^	Sri Lanka	Prevention of reintroduction	Surveillance response Vector control
Mercado et al.^17^	22 countries in Asia		Surveillance response National malaria policies
WHO^31^	South-East Asia		Border areas
Perera et al.^18^	All countries		Surveillance response
Ranaweera et al.^33^	Sri Lanka	Prevention of reintroduction	Other emerging communicable diseases
Teo et al.^28^	Asia Pacific region, Singapore for malaria example	Prevention of reintroduction	Transfusion Malaria
Voumard et al.^26^	Switzerland		Travel as a risk factor
Wang et al.^27^	Taiwan	Prevention of reintroduction	Vector control Competing interests

### Surveillance and response.

Sri Lanka, Saudi Arabia, and Greece have eliminated or nearly eliminated local transmission but now face continuous threats of importation and reestablishment at their borders. In terms of surveillance and response, Saudi Arabia focuses specifically on Hajj; Greece has high rates of immigration and focuses on controlling resurgence; and Sri Lanka focuses on maintaining vigilance, fast containment, and monitoring foreign workers.^11^^–^^14^^,^^16^

Even when surveillance systems are established in countries, sometimes the processes of data collection and reporting are disorganized and incomplete. In 22 countries in the Asia Pacific region, surveillance data were incomplete and not comprehensive enough to facilitate effective responses.^17^ Most malaria incidence data collected by National Malaria Control Programs originate from government-operated health facilities, but not all populations at risk for malaria have access to these facilities, and many malaria patients seek treatment in the private sector or from other service providers. Many National Malaria Control Programs in Asia Pacific did not collect data from mobile and migrant populations, the private sector, or the military.^17^ Additional challenges include paper-based information flow and data collection systems, particularly at the community level, and the recommendation of resource intensive, individual case-based surveillance and response strategies that many countries lack capacity to adopt and implement well.^18^

### National malaria policies.

Establishing national policies and implementing them are important for both achieving and maintaining malaria elimination and strengthening global health security. Although clear national policies are important for all aspects of malaria program operations, the overlap with health security is illustrated in the areas of treatment, including regulation of the private sector, and migrant health.

Treatment guidelines form an essential health sector intervention to augment health security. In certain countries (Cambodia, Lao PDR), the private sector primarily comprises general retailers, itinerant drug vendors, and pharmacies; these providers lacked supervision and training to carry out testing. Often, antimalarials are available in private pharmacies and in private shops and are dispensed without a malaria diagnosis.^17^^,^^19^ Private sector outlets often use drugs that are not in the national treatment guidelines; most outlets are low on first-line treatment and stock more second-line treatment.^17^

Migrants are a potential threat for the spread of drug-resistant malaria and a barrier for malaria elimination.^20^ Countries in the Greater Mekong Subregion have specific laws to safeguard the health and welfare of migrant and foreign workers, including conditions for employers to fulfill in relation to malaria and its control.^21^ Several such laws exist in Lao PDR, but implementation and enforcement remain a challenge. Worksites are generally remote and lack access to health, education, and basic services, and affected workers are likely to be poor, illiterate, and belong to an ethnic minority group.^21^ Development of specific information, education, and communication, such as roadside billboards targeting migrant workers and mass media campaigns, are part of the malaria strategy.^20^

In the Greater Mekong Subregion, migrants are a known high-risk population for malaria transmission, but their rights and access to healthcare is variable by country; this will have an impact on each country’s ability to eliminate, and the fluid borders mean that all countries are likely to be affected. In Thailand, the Workmen’s Compensation Act protects migrants against work-related injury in workplaces with 10 or more employees.^20^ In Cambodia, the Labor Law covers all workers regardless of their nationality except domestic or household aides.^20^ Vietnam has few labor regulations ensuring health rights for migrant workers.^20^ Myanmar’s labor laws are more than 60 years old and are limited in scope; they do not provide health rights for migrant workers.^20^

### Travel as a risk factor.

Travel has been described as a risk factor for control and elimination of malaria.^20^ Travelers from malaria-endemic countries visiting malaria-free areas can initiate a cycle of transmission in highly receptive areas, and travelers from malaria-free areas visiting malaria-endemic areas can bring the malaria parasite to malaria-free areas. In Greece, where malaria was previously eliminated, the disease was reintroduced by way of migrant army workers, which led to local transmission and outbreaks.^12^ Security forces returning from foreign deployment in malaria endemic countries (e.g., United Nations peacekeeping forces) have posed a serious threat of reestablishment of the disease in Sri Lanka.^24^

Pretravel medical advice for overseas travelers is provided in some countries.^23^^,^^25^ In Sri Lanka, pretravel advice and chemoprophylaxis are provided free of charge to travelers visiting malaria-endemic countries.^13^ In a Kenyan study, 70.6% of respondents knew that some health services are provided to international travelers^25^ In Taiwan, among 223 imported malaria cases reported between 2002 and 2013, more than 60% of the travelers had not received pretravel advice.^23^

Risk perception is another determinant of utilization of services and compliance to advice. In Switzerland, 85% of travelers to low- to moderate-risk malaria areas chose not to take chemoprophylaxis as malaria prevention, although most guidelines recommend it.^26^ Among Sri Lankan security personnel traveling to malaria endemic countries on UN peacekeeping missions, the majority were well aware of the need to start chemoprophylaxis before departure and the need for its regular use as prescribed during the overseas stay; however, more than 80% were unaware that the medication had to be continued for a specified period of time after their return. In the same study, the overall adherence to chemoprophylaxis was 78.7%; malaria chemoprophylaxis leading to impotency has been cited as a reason for poor compliance.^24^ Some travelers have coherent reasons for their choice; new recommendations should include shared decision-making to take into account travelers’ preferences.^26^

Migrants who travel across borders often represent vulnerable populations, fleeing economic hardship or civil or social disruption; they generally stay under the radar and are often a difficult group to target in prevention interventions.^22^

### Vector control.

Vector control continues to be an important intervention to prevent reestablishment of transmission after countries achieve elimination. Mosquito management programs featured prominently in the control of reestablishment of malaria in Greece.^12^ In Taiwan, environmental management and vector-control efforts were used to prevent outbreaks and sustain malaria elimination.^27^ In Sri Lanka, indoor residual spraying and larvicides continue to be used as vector-control methods after malaria-free certification.^16^

### Transfusion malaria.

Preventing transfusion malaria was a priority in Greece when the first locally acquired malaria cases were reported in 2011. A directive, based on EU guidelines, included deferral from blood donation for a period of 6 months for persons residing or working in the affected areas within a radius of 10 km from the residence of locally acquired cases. One of the main criteria included in the EU directive is that the deferral applies also to all persons who have “visited endemic areas.”^21^ In the Asia Pacific region, current donor selection criteria are only partially effective in excluding infected donors and are unable to detect semi-immune and infected individuals in whom the parasite may persist for long periods of time with no symptoms.^28^

### Competing interests.

As the incidence of malaria cases declines, countries tend to focus attention on other health priorities and become complacent, resulting in lack of preparedness for malaria outbreaks. In Nepal, as the number of malaria cases decreased, attention towards prevention also decreased; imported malaria cases were not investigated and regular meetings for malaria prevention were not held.^29^

When a country achieves or is on the verge of malaria elimination, the disease is likely to be forgotten by clinicians and administrators, and surveillance is likely to be threatened.^30^ Clinicians forget to ask the important question of a travel history in the recent past in a febrile patient; instead, the patient is subject to a plethora of investigations. The differential diagnosis of fever in a person who has returned from travel should always include malaria as one of the primary possibilities. Wang et al. stressed the need for clinicians and healthcare workers in Taiwan, a malaria-free but a receptive and vulnerable country, to continue to report all malaria cases to their respective county public health authorities.^27^ Administrators and policy makers find it difficult to justify malaria budgets with a declining malaria incidence, and this often leads to cuts in financial allocations. In Sri Lanka, such a situation arose when the number of reported cases in 1963 was only 17, of which 11 were imported^30^; this was followed by a resurgence that took another 50 years to eliminate the disease.^13^

The perception that malaria is not a healthcare problem during elimination and the prevention of reestablishment phases needs to be countered.^30^ Declining perceived risks associated with a decreasing incidence leads to cuts in financial allocations for disease control activities, resulting in out-of-pocket expenses for patients and vulnerable populations mostly having to depend on a poorly regulated private sector.^29^

### Border areas.

Health and other complementary social/welfare services along international borders are typically weaker and more poorly staffed than in more central areas, in part because some of these areas may be chronically affected by security concerns and tensions. Controlling illegal migration across borders is an important aspect of malaria control in border regions. Moreover, many of the people living in border areas, especially in remote ones, are from socioeconomically vulnerable minority groups.^22^ Such people are often disadvantaged in terms of access to healthcare and social services, and in numerous instances they lack citizenship rights.^31^ These major structural constraints affect all aspects of malaria elimination efforts, including timely prevention, diagnostic testing and treatment, surveillance, monitoring and evaluation, epidemic forecasting, and rapid response capacity to prevent and contain outbreaks and resurgences.^31^ Continued commitment from both sides of the border is required to curb disease spread.^22^

Widely differing treatment regimens and vector-control practices, and timelines of implementing interventions in neighboring countries is a major problem in the border areas of the Southeast Asia region. Timely epidemiological data on the malaria situation in border areas is incomplete or even absent, although it is a component of IHR that all member states should report to the WHO. Another chronic challenge is posed by limited intercountry, cross-border cooperation and collaboration on malaria elimination, particularly in sensitive disputed border areas.^19^^,^^31^

During discussions over malaria control on the Cambodia and Vietnam border, participants were of the consensus that the IHR 2005 was an effective instrument to mandate global notification of health emergencies of international concern; however, lack of a clear legal framework to support regional programs was seen as a barrier to the establishment of direct communication across borders.^15^ Moreover, the movement of data and information from production sites to other places can be challenging because of different standards and practices, language barriers, different national structures and rules that govern the circulation of health information inside and outside countries, imbalances in capacities and power, and sustainability of financing arrangements.^15^

### Other emerging communicable diseases.

Emergence of other communicable diseases that pose a more acute threat to population health can overshadow malaria risk and its future control. Those who interact with malaria patients now face another risk of contracting COVID-19 while trying to rule out malaria infection in a suspected patient. Such workers should be provided with personal protective equipment while handling febrile patients.^32^ In addition, efforts to contain the spread of COVID-19, such as international border closures and lockdowns, led to rapid repatriation of persons from malaria-endemic countries, a potential threat to eliminate or prevent the reestablishment of malaria.^33^

## DISCUSSION

The IHR were established to ensure safe global travel and trade while preventing disease spread across borders. To successfully implement the recommended regulations, controlling a disease within the country is a prerequisite; malaria programs in the control phase do not differentiate between locally transmitted and imported malaria cases until they reach the elimination phase. Hence, the papers included in this review primarily focus on how health security issues related to international travel may affect elimination and prevention of reestablishment (POR) of malaria programs.

Increased and improved surveillance, one of the capacities that countries are expected to achieve to ensure regional and global health security goals, plays a major role in containing diseases. Surveillance, including making malaria a notifiable disease, has been recognized as a pillar of the GTS, but not all malaria-endemic countries have invested in doing so.^7^ Different malaria surveillance modalities are practiced in different countries and regions to ensure prompt detection and response, but their completeness and comprehensiveness vary. Lack of financial resources, trained staff and other resources, and clear guidelines have a negative impact on the effectiveness of surveillance and response strategies and activities.^18^ Absence of data from military and mobile populations, major risk groups for malaria, is a limitation of surveillance systems, especially in border regions where illegal migration occurs. Cases undetected by the system can be a threat when the country is trying to reach elimination targets. Differences in socioeconomic conditions and the use of different drug regimens across borders, as well as the lack of coordinated control activities, may contribute to the mounting problem.

Clearly defined and properly enforced policies can play a major role in controlling diseases. Malaria has the potential of escalating into epidemic proportions, and therefore countries should regulate malaria diagnostic and treatment services. Involving both the public and private sectors and even NGOs in service provision has advantages in being able to reach a wider population, but all providers must be held accountable to national policies and guidelines.

Comprehensive data collection and sharing is important in evaluating the disease burden and success of control and elimination strategies. It also helps to compare situations in different countries. This is especially important in landlocked countries where border malaria is a problem. However, this review highlights several important issues that have arisen due to inconsistent and incomplete data collection, standards, and ineffective communication that are important under the IHR. The problems include inconsistencies in case definitions that make it difficult to compare cases, low reliability of information systems, trustworthiness of shared information, and politics of participation at cross-border regional meetings where different levels of technical proficiency and ownership of data and data collection systems impact on the ability of country partners to share data and speak confidently. The imbalances in capacities reflect the influence on the dynamics of data and information sharing. Cross-border regional meetings provide an opportunity to initiate or consolidate personal relations between health professionals in different countries, which can be used to promote informal communications in the event of disease outbreaks or other public health needs. Regional networks such as ASEAN may be used as an overarching framework to coordinate regional public health initiatives as part of the IHR.^15^

The countries that are close to eliminating malaria or have already achieved elimination face unique challenges, especially when they are receptive and vulnerable to reestablishment of transmission. Ongoing training and communication of diagnosis and treatment guidelines for clinicians and other providers must be prioritized even as malaria incidence declines to zero to avoid complacency and ensure rapid detection of imported or transfusion-based malaria cases. Targeted vector control, one of the pillars of the GTS, must also be maintained in specific contexts after elimination as long as malaria vectors are still present or risk being introduced.^12^^,^^16^^,^^27^^,^^33^ When malaria is endemic in neighboring countries, reestablishment is always possible by way of legal and/or illegal border crossings especially when land borders exist. Migrant workers are a vulnerable community due to unfavorable working and living conditions and lack of access to healthcare; they represent an important source of maintaining malaria transmission and facilitating its reestablishment. Many countries use migrant or foreign labor that may come from malaria-endemic countries. According to the International Labor Organization Conventions, countries hosting migrant workers should have labor and social laws that protect their rights, especially concerning working conditions and occupational health and safety. Migrant workers should be provided with access to health services, including access to emergency care, similar to the nationals of the country.^20^ Surveillance systems should be expanded and enhanced to detect infections among mobile populations and the country’s health system, and laws should recognize vulnerable mobile populations and foreign workers.

Other health concerns, such as the current COVID-19 pandemic, have been shown to affect malaria control and elimination. In the past 2 years, public health systems have focused on the control of the COVID-19 pandemic with all other diseases being largely neglected. In the early stages of the pandemic, chloroquine, an antimalarial drug, was proposed to be effective against COVID infection but later disproved. If widespread use of the drug for treatment of COVID had occurred, it may have increased resistance in malaria parasites, undermining global malaria elimination efforts.^32^ Sri Lanka experienced a situation in which a large number of repatriates from malaria-endemic countries were brought back to the country from time to time; because the country is receptive, the Anti Malaria Campaign (AMC) had to be extra vigilant and screened all repatriates from malaria-endemic countries at quarantine centers. If the quarantine center was located in a previously malaria-endemic area, entomological surveys were conducted around the quarantine center; if malaria vectors were found, a preemptive vector control program was implemented promptly. Although the expertise and resources of the AMC were not directly used for the COVID-19 response, the Sri Lankan example highlights the importance of a highly coordinated program between multiple sectors and departments that were involved in the COVID-19 response and the malaria POR program, which is an integral part of IHR. It also demonstrates the importance of a sound public health infrastructure with adequate, trained field staff for contact tracing and testing, which was the common factor for the success of both malaria elimination and POR and the effective COVID-19 response in Sri Lanka.^33^

The ambit of IHR has still not included and integrated malaria. With many countries targeting malaria elimination in the future, aligned with the goals of the GTS, application of IHR is a must for malaria elimination and preventing reestablishment to ensure achievement of global goals.

Given that the IHR focuses on prevention of cross-border spread of infectious diseases, malaria surveillance and response are a key strategy as outlined in the GTS.^7^ Blood safety may also be considered a part of the IHR if there is a risk of disease transmission once eliminated.

There are several aspects to consider in implementing the IHR.^3^ The most important actions to be considered include strengthening surveillance and response; understanding the WHO’s role in the IHR; increasing public health security at ports, airports, and ground crossings; and disseminating and sharing timely data. Given these activities, malaria control programs targeting elimination and in prevention of reestablishment phases can easily use an IHR model, especially in border areas, as most of the recommendations in the GTS relate to these aspects. Potential actions that national malaria programs in different phases of control can consider to facilitate integration of implementation of relevant aspects of IHR are listed in Table 3. These actions primarily focus on universal health coverage for diagnosis and treatment and improvement of surveillance systems that are key strategies to preventing further spread of the disease.

**Table 3:** Potential actions that can be taken by national malaria programs in different phases of malaria control to facilitate integration and implementation of the International Health Regulations (IHR)

IHR domain	Control phase	Elimination phase	Prevention of reestablishment phase
Know IHR: purposes, scope, principles and concepts	✓ Train staff	✓ Ensure political and financial commitment	✓ Ensure political and financial commitment
		✓ Train staff	✓ Train staff
Update national legislation		✓ Make malaria a notifiable disease ✓ Ensure case and foci investigation	✓ Ensure case and foci investigation
Recognize shared realities and the need for collective defenses	✓ Multisectoral collaboration	✓ Multisectoral collaboration	✓ Multisectoral collaboration
Notify, report, consult and inform WHO	✓ Improve surveillance systems	✓ Improve surveillance systems	✓ Improve surveillance systems
Improve national surveillance and response capacities	✓ Universal access to diagnosis and treatment services to all, including migrants	✓ Universal access to diagnosis and treatment services to all, including migrants.	✓ Universal access to diagnosis and treatment services to all, including migrants ✓ Maintain strict vigilance
		✓ Improve surveillance system—conduct case and foci investigation and classification	✓ Conduct case and foci investigation and classification
		✓ Establish rapid response teams	✓ Maintain rapid response teams
	✓ Training of staff on IHR and malaria control	✓ Training of staff on IHR and malaria elimination	✓ Training of staff on IHR and prevention of reestablishment of malaria
Increase public health security at ports, airports and ground crossings		✓ Have malaria diagnosis and treatment facilities	✓ Have malaria diagnosis and treatment facilities
	Share data with neighboring countries	Share data with neighboring countries	Share data with neighboring countries

## CONCLUSION

Malaria has not been prioritized as part of the IHR, nor has the IHR focused on vector-borne diseases such as malaria. The IHR in its current format can be applied to malaria and other vector-borne diseases but may require some additional guidelines. Using the IHR framework will facilitate overcoming some of the issues of complementary actions at borders and data and information sharing, especially among countries targeting elimination. With global elimination goals, the application of IHR for malaria should be urgently reviewed and included as part of IHR.
